# First Detection of Hepatitis E Virus RNA in Ovine Raw Milk from Herds in Central Italy

**DOI:** 10.3390/foods13203218

**Published:** 2024-10-10

**Authors:** Gianluigi Ferri, Luca Pennisi, Filiberto Malatesta, Alberto Vergara

**Affiliations:** 1 Department of Veterinary Medicine, Post-Graduate Specialization School in Food Inspection “G. Tiecco”, University of Teramo, Strada Provinciale 18, 64100 Teramo, Italy; lmpennisi@unite.it (L.P.); avergara@unite.it (A.V.); 2 Food and Health Veterinarian Consulting, 64100 Teramo, Italy; izv.filiberto@virgilio.it

**Keywords:** hepatitis E virus, RNA, ovine, raw milk, molecular biology, food safety, public health

## Abstract

HEV mainly enters animal and human hosts through the orofecal route, which presents a critical health concern alongside the associated environmental variable. Among products of animal origin, milk (both ovine and bovine) can harbor HEV RNA, which can potentially be transmitted to consumers. In this study, a total of 220 raw ovine milk samples were collected from Apennine breed subjects farmed (transhumance method) in three different Italian provinces, L’Aquila, Pescara, and Teramo, located in the Abruzzo region (Central Italy). All the specimens were screened using one-step real-time RT-qPCR and nested RT-PCR assays. Among them, 5/220 or 2.27% harbored HEV RNA fragments belonging to the ORF1 and ORF2 codifying regions of the genotype 3c. The average viral amount discovered was 10^2^ GE/mL. These subjects represented 2/57 or 3.51% of the Pescara herd, and 3/105 or 2.86% of the Teramo herd. Although HEV RNA was discovered in sheep fecal samples, the original data obtained in the present study represent the first HEV RNA detection in ovine raw milk from Italy.

## 1. Introduction

Among foodborne pathogens (e.g., hepatitis A virus and norovirus), hepatitis E virus (HEV) has attracted greater attention due to its environmental persistence, which has contributed to its definition as an emerging and remerging viral *noxa* [1]. Indeed, it is considered responsible for hospitalizations in developing countries, as it is mainly related to the circulating genotypes (HEV1 and HEV2) that infect humans through the ingestion of contaminated water. Conversely, HEV3 and HEV4 sporadically cause gastrointestinal symptoms and are mainly dispersed throughout developed countries. However, weaker human consumers can become receptive and symptomatic to HEV3 and HEV4, representing a crucial public health concern [1].

Based on its structural characteristics, it is classified as a quasi-enveloped virus, which confers high resistance to various challenging physical and chemical conditions, such as extreme acid solutions (pH < 2.0), high osmolarity, etc. [2,3]. Alongside these properties, HEV has ensured its evolutional survival by adapting and introducing itself to both domestic and wild life cycles. Indeed, many mammalian species are viable hosts, including humans [4].

Among the eight genotypes discovered, HEV3 is widely circulated throughout the European continent, and its targets, food-producing animal hosts, include the *Suidae* family (especially wild boars and domestic pigs) and small ruminants (ovine, caprine, wild) [5]. HEV diffusion among domestic and wild animals mainly occurs through fecal diffusion and is bolstered by environmental sharing; humans are infected through the ingestion of raw or under-cooked contaminated foodstuffs originating from viremic animals (i.e., fresh meat or liver sausages, raw milk, raw mussels, etc.) [6,7,8,9].

Among animal products, the milk food matrix was recently found to be an HEV transmission route. Indeed, RNA fragments (two overlapping open reading frames (ORFs) such as ORF1 and ORF2) were amplified from raw and pasteurized specimens collected from bovine, ovine, and caprine species [10], as well as humans [11]. The physiopathological explanation points to interaction between host cytotype receptors (syndecans) and the HEV capsid ligand, which was demonstrated by Singh et al. to be the pORF2 protein [12]. This implies that HEV virions can enter the mammary gland during the viremia phase and express adenomeres on the above-mentioned intermembrane receptor [12]. As such, the possibility of consumer infection through the ingestion of HEV-contaminated raw milk, derived from all dairy-producing animals including ovine animal species, emerges. Indeed, in Europe, HEV RNA has rarely been amplified in milk specimens, with a 2.80% prevalence observed by Dziedzinska et al. [13] in the Czech Republic and a 12.30% prevalence described by Demirci et al. [14] in ovine species in Turkey. On the other hand, HEV RNA has not been discovered from bovines, as reported by Vercouter et al. [15] in Belgium and Baechlein and Becher [16] in Germany. In Italy, HEV genome and genome segments have never been amplified in sheep or dairy cattle milk samples, and there are currently no confirmed infection cases related to the human consumption of ovine raw milk. Its detection could have sanitary repercussions, especially for specific production chains, such as raw milk cheese, and for unpasteurized or non-boiled products. Viable HEV forms (during viremia in receptive animal hosts), dispersed through milk, represent a focal public health concern, especially for consumers with compromised health (e.g., HIV-infected patients, transplant recipients, etc.). Furthermore, the efficacy of thermal treatment, such as pasteurization, hugely depends on the initial viral loads harbored by foodstuffs [1].

Based on this reasoning, the present study aimed to find HEV RNA in unpasteurized ovine milk specimens, using quantitative (one-step real-time RT-qPCR) and qualitative assays (nested RT-PCR). Samples were collected from 220 extensively farmed subjects (Apennine breed), following the traditional transhumance method, in three different Italian provinces (L’Aquila, Pescara, and Teramo) located in the Abruzzo region. Another driver of this study is the possible peculiarity of environmental sharing between the studied animals and wild ones, as the explored grazing areas include three national parks that are normally populated by both reservoirs and/or receptive hosts (wild ruminants).

## 2. Materials and Methods

### 2.1. Samples Collection

**Figure 1:**
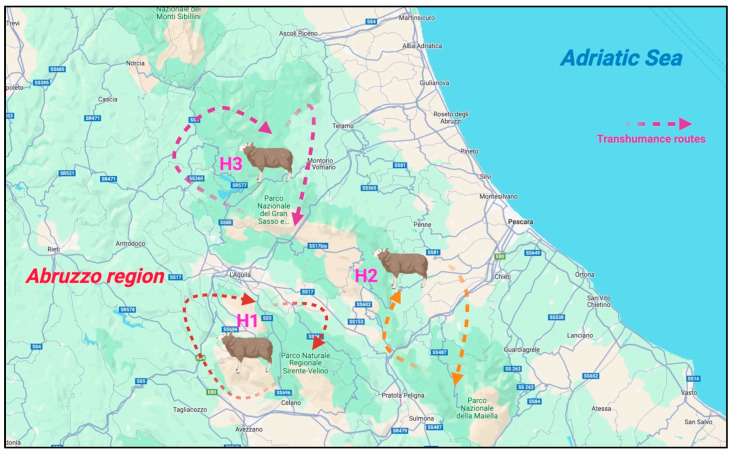
Animal herd distributions and the respective transhumance routes of the screened subjects in the Abruzzo region, Central Italy.

Between January and February 2024, a total of 220 Apennine breed sheep (*Ovis aries*) were involved in the present scientific investigation. In detail, the animals were extensively farmed, following the transhumance method, in three different National Parks (Gran Sasso, Maiella, and Sirente Velino), located in the provinces of L’Aquila, Pescara, and Teramo (Abruzzo region, Central Italy) (See Table 1). The Abruzzo region was included due to HEV RNA circulation being discovered in the feces of small ruminants (ovine), as well as high IgG titers being found their sera in 2019 [17]. The scientific hypothesis of milk excretion is also supported by the biochemical evidence described by Singh et al. [12], who confirmed possible human infection. A detailed illustration of the herds and their respective transhumance routes in the three screened provinces is shown in Figure 1.

These zones cover wide surfaces: 1413 km^2^ for Gran Sasso National Park, 628.4 km^2^ for Maiella National Park, and 543.6 km^2^ for Sirente Velino. The adopted farming method (transhumance) represents a rooted form of traditional ovine management, which permits animals to feed on seasonal fodder for grazing, as suggested by Serranito et al. [18].

In further detail, the areas used for animal feeding are normally populated by wild animal species, i.e., wild boars (*Sus scrofa*) and ruminants (*Rupicapra pyrenaica ornate*, *Capreolus capreolus*, etc.), characterized by an average density of 3.8/100 ha [19].

The screened ovine population comprised pluriparous and primiparous sheep with an average age of 2.5 ± 0.8 years and a weight ranging between 45 and 55 kg. The target food matrix for HEV RNA detection was raw (unpasteurized) ovine milk collected from the subjects, more specifically, during lactation peaks. During each sampling step, pre- and post-dipping procedures were performed, first by cleaning the udders, which were successively disinfected with chloride-based disinfectant solutions. Final collected volumes of 50 mL were gathered individually from each animal. All the specimens were then transported under refrigerated conditions directly after collection and immediately processed at a laboratory level.

### 2.2. Samples Processing and RNA Extraction

Starting with the initial volume of 50 mL collected from each animal, 2.5 mL of unpasteurized milk was spiked with 5 × 10^6^ MS2-phage-like particles (MS2-PLP) to allow for efficacy evaluation of the performed screening, as described by Mikel et al. [20]. The 2.5 mL/subject aliquots were involved in a first separation step for fat layer stratification, which was performed by centrifugation at 3000× *g* for 15 min at 4 °C. At the end of this step, this layer was removed. Milk protein and possible viral particle precipitation was facilitated by acidification using an HCl solution 1M (pH 3.5–4.0); in detail, 300 µL of the solution was added to each specimen (2.5 mL/subject). A second centrifugation was performed at 4000× *g* for 10 min at 4 °C, and the supernatant was subsequently removed. The obtained pellets were resuspended in 5 mL of PBS (pH 7.2) (ThermoFisher Scientific^TM^, Milan, Italy), as reported by Dziedzinska et al. [13]. The second step was an RNA extraction phase, which was conducted following the TRIzol LS method (Invitrogen, Ltd., Paisley, UK). For this, the two reagents TRIzol (250 µL/specimen) and chloroform (100 µL/specimen) were added to an initial aliquot of 500 µL (v1), collected from each processed milk sample (as previously described). After centrifugation at 12,000× *g* for 15 min, the supernatant was removed, and the obtained pellet was washed with 500 µL of isopropyl (75.0%) and ethylic alcohols (75.0%). A final centrifugation at 7500× *g* for 5 min was performed, and the final pellet products were resuspended using 50 µL of sterile RNase-free water (Invitrogen UltraPure DNase/RNase-Free Distilled Water, ThermoFisher^TM^, Waltham, MA, USA). All the extracts were finally stored at −80 °C until biomolecular screening.

### 2.3. Real-Time RT-qPCR and Nested RT-PCR Assays

Biomolecular assays begin with quantitative tests performed using one-step real-time RT-qPCR, and the GENE UP^®^ System (bioMérieux, Paris, France) was used for HEV RNA genome equivalent/mL (GE/mL) detection. In further detail, specific commercial kits (ceeramTools—Thermo Fisher Scientific^TM^, Waltham, MA, USA) for HEV RNA detection were used. All the final reaction volumes were 25 µL, comprising 20 µL of reagents and 5 µL of templates (extracted RNA); positive and negative controls were included, following the manufacturer’s instructions (ceeramTools—Thermo Fisher Scientific^TM^, Waltham, MA, USA). Standard curves of known concentrations (from 10^7^ to 10^3^ GE/mL) were used to determine the quantitative nucleotide concentrations (expressed as GE/mL). The fluorescence channel parameter associated with each well was 520 nm. The so-called limits of detection (LOD) and quantification (LOQ) were revealed by performing 10-fold serial dilutions ranging from 10^7^ to 10^3^ GE per 2.5 mL of sample. Furthermore, the lowest concentration of MS2-PLP was represented by the LOD parameter, which was considered to be detectable with a probability of 100%; finally, the LOQ value described the smallest amount of analyte (coefficient of variation of 25%), as reported by Kralik and Ricchi [21]. The thermocycler was set as follows: initial reverse transcription at 45 °C for 10 min; denaturation at 95 °C for 10 min; and 40 cycles at 95 °C for 15 s, 60 °C for 45 s, and 72 °C for 45 s, in agreement with the manufacturer’s instructions (ceeramTools—Thermo Fisher Scientific^TM^, Waltham, MA, USA).

Nested RT-PCR assays were performed as qualitative confirmation of the positive subjects in one-step real-time RT-qPCR, amplifying specific genetic regions of the HEV genome and, more specifically, ORF1 and ORF2. Furthermore, the primer couples HEV-cs and HEV-cas for ORF1, as described by Johne et al. [22], and HEV-ORF2con-s1 and HEV-ORF2con-a1 for ORF2, as designed by Wang et al. [23], were used in the first reaction (reverse transcription). This was achieved using the Qiagen^®^ OneStep RT-PCR Kit (Hilden, Germany) with final volumes of 20 µL and 5 µL of extracted RNA. The second step (nested PCR: HEV-csn and HEV-casn for ORF1; HEV-ORF2con-s2 and HEV-ORF2con-a2 for ORF2) was performed using Green Master Mix Promega^®^ (Madison, WI, USA) kits with final reaction volumes of 25 µL, including 1.5 µL of amplicons that were obtained from the reverse transcription (first) step. A positive (ATCC^®^ VR-3258SD RNA fragment, Manssas, VA, USA) and negative control (sterile RNase-free distilled water, ThermoFisher^TM^, Waltham, MA, USA) were included in each qualitative assay.

The thermocycler settings and respective amplicon seizures were set as in the respective references [22,23].

The nested RT-PCR products were finally loaded onto agarose gels at different concentrations (1.5% and 2.0%) depending on size, and gel running was performed at 80 V for 40 min. The expected positive bands were compared with specific DNA ladders (50 bp and 100 bp) (Genetics, FastGene^®^, Düren, Germany). We expected positive fragments (nested RT-PCR products) of 145 bp for the ORF2 genetic determinant, as described by Wang et al. [23], and 333 bp for ORF1 [22]. All the suspected fragments were purified using the commercial Qiagen QIAquick^®^ PCR Purification Kit (Hilden, Germany) and sent to Biofab Laboratories (Rome, Italy) for sequencing following the Sanger method. Sequences were used for nucleotide similarity analyses performed using the BLASTN system (https://blast.ncbi.nlm.nih.gov/Blast.cgi?PROGRAM=blastn&BLAST_SPEC=GeoBlast&PAGE_TYPE=BlastSearch) (accessed on 12 June 2024). They were also loaded and evaluated using the HEV typing tool (https://www.rivm.nl/mpf/typingtool/hev/, accessed on 3 July 2024), as reported by Baylis et al. [24]. The obtained sequences were loaded and registered on the GenBank platform (https://www.ncbi.nlm.nih.gov/genbank/, accessed on 3 July 2024).

### 2.4. Statistical Analysis

Statistical analysis was performed using IBM^®^ SPSS 20.0 software (SPSS, Chicago, IL, USA). The two-tailed *t*-test was selected for reference, and the dependent variable was HEV amount, in GE/mL, which was compared based on the respective geographical areas. For the descriptive statistics, the normality assumption was evaluated with the Shapiro–Wilk test, and the alpha was <0.05. The calculated percentages also included the confidence interval (CI) at 95%, when applicable.

## 3. Results

A total of 220 female ovine (Apennine breed) subjects were investigated in the present scientific investigation through the performance of biomolecular assays (HEV RNA detection) involving unpasteurized milk specimens.

Using one-step real-time RT-qPCR and nested RT-PCR analyses, HEV RNA was discovered in 5/220 or 2.27% (CI 95%: 0.31–4.23%) of the tested milk samples. Based on the herd distributions, three out of the five positive subjects belonged to H3, representing 3/105 or 2.86% (CI 95%: 0.01–5.71%), with 2/57 or 3.51% from (CI 95%: 0.09–6.93%) H2. HEV RNA was never amplified in H1. The results of the one-step real-time RT-qPCR (expressed as GE/g) are schematically reported in Table 2.

The performed *t*-test revealed statistically significant differences in the HEV RNA amounts (GE/g) between H2 and H3 (*p*-value < 0.02).

**Figure 2:**
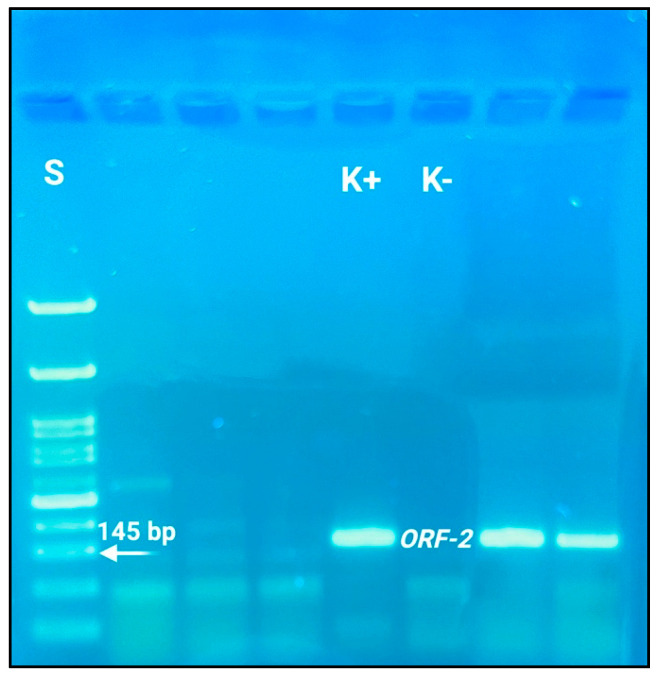
Electrophoresis assay relative results (agarose gel 2.0%), showing positive nitid bands belonging to the nested PCR product (145 bp) genetic determinants of ORF-2. S: standard (DNA ladder 50 bp (Genetics^®^ Fast Gene, Düren, Germany)); K+: positive control; K−: negative control.

The qualitative nested RT-PCR assays mainly amplified ORF-2 genetic determinants (compared to ORF-1); Figure 2 shows the nitid amplicons (145 bp) obtained by the second reaction.

The results of the Sanger sequencing (described in the Section 2) were successively analyzed using the BLASTN system (https://blast.ncbi.nlm.nih.gov/Blast.cgi?PROGRAM=blastn&BLAST_SPEC=GeoBlast&PAGE_TYPE=BlastSearch, accessed on 12 June 2024) and HEV typing tool (https://www.rivm.nl/mpf/typingtool/hev/, accessed on 3 July 2024), as described in the Materials and Methods. Sequences were registered on the GenBank platform with the following accession numbers: PQ043349, PQ043350, and PQ043351 for the ORF2 fragment (145 bp); PQ043352 and PQ043353 for the ORF1 amplicon (333 bp) (See Table 3).

Both the BLASTN and HEV typing tool alignments revealed high nucleotide similarities (100.0%) to the HEV genotype 3c for all positive amplicons. Additionally, all the obtained sequences shared 96.0–98.0% nucleotide identity between them. The highest (99.0%) similarity for ORF1 among the discovered sequences was amplified from hunted wild boars’ (*Sus scrofa*) liver and muscle tissues in the Marche region (Central Italy), registered with the accession numbers MN20202101, MN20202102, and MN20202103 [25].

All of the ORF1 sequences presented high nucleotide similarity (nt. ID over 98.0%) with previously deposited GenBank sequences.

Parts of HEV ORF2 were observed in both H2 and H3, as illustrated in Table 3; among them, the registered PQ043349 (positive raw milk sample collected from H2) presented the highest nucleotide similarity (nt. ID 100.0%) to NC_ 074955.1 and JF443720, which were obtained from HEV-infected human patients [26,27], and AH006999, derived from raw sewage in Spain and discovered Pina et al. [28]. PQ043350 was similar (nt. ID 95.24%) to MZ814707, which was amplified from immunocompromised HEV3c-infected human patients that presented gastrointestinal symptoms, such as nausea and diarrhea, and hepatic necrotic focal lesions in Germany, as described by Schemmerer et al. [29]. Finally, PQ043351 was identical to HEV3 discovered in swine stool (EF681109) (more specifically, cecal content [30]) and human sera (MG020088) from the blood of HEV-infected patients (with bimolecular HEV confirmation) [31].

## 4. Discussion

Based on the ultrastructural characteristics of HEV (classified as quasi-enveloped) virions, it presents well-known evolutionary adaptation to both marine and terrestrial environments. Indeed, HEV can also survive and persist in different ecosystems thanks to reservoir or spill-over from domestic and wild animal species, as described by Primadharsini et al. [32].

Furthermore, its environmental behavior has further indicated cross-species infections, which especially increase through environmental sharing with spillover hosts (especially wild boars and ruminants) receptive to this pathogen [33].

From a physiopathological perspective, during viremia, HEV gains different target cytotypes, such as hepatocytes, mammary adenomeres, etc. The potential for infectious virion excretion through the milk matrix has been widely demonstrated in both dairy animals and humans, with high HEV titers observed during lactation peaks. In such instances, the combination of high mammary blood perfusion and viremia facilitate virion diffusion and execration, making raw milk a contaminated food matrix able to infect different receptive hosts [12].

HEV is an enteric foodborne pathogen, and it is mandatory to consider possible human infections through the ingestion of contaminated milk. This risk is further heightened by the variable efficacy of pasteurization, which depends on the initial viral loads in raw milk and its derived products. It implies several possible sanitary repercussions for consumer health, especially in immunocompromised individuals, as suggested by Huang et al. [34].

Following this rationale, the present study aimed to biomolecularly screen pasteurized milk specimens collected from 220 extensively farmed (transhumance) Apennine breed ovines in the Abruzzo region (Central Italy), applying quantitative and qualitative methods, performing one-step real-time RT-qPCR and nested RT-PCR. Indeed, the three studied herds grazed in three different provinces (H1: L’Aquila; H2: Pescara; and H3: Teramo, as previously indicated in Table 1), which, more specifically, are included in three national parks (Gran Sasso National Park, Maiella National Park, and Sirente Velino).

As previously described in the Section 2, these parks are the habitats of many HEV-receptive reservoir (especially wild boars) animal species. Therefore, the scientific hypothesis behind possible HEV RNA circulation in ovine raw milk specimens, based on the particular environmental conditions of ecological sharing of these parks, drove the present investigation to provide original epidemiological data and also focus on possible infection from contaminated raw milk consumption.

Among the screened animals, 5/220 or 2.27% (CI 95%: 0.31–4.23%) harbored genetic target sequences belonging to the ORF-1 and ORF-2 codifying HEV genomic regions. The obtained prevalence value is in line with the 2.80% value reported by Dziedzinska et al. [13] for raw ovine milk specimens in Europe (Czech Republic). The one-step real-time RT-qPCR results in this study revealed average values of 10^2^ GE/mL, similar to the amounts observed by Dziedzinska et al. [13]. In addition to exploring positive sheep, they also amplified HEV RNA from goats. The HEV RNA amounts found in both studies (10^2^ GE/mL) cannot induce symptomatic expression in immunocompetent human subjects (infectious dose: 10^5.5^ GE/mL), and based on genotype 3’s virological characteristics, it sporadically causes hospitalizations, as reported by La Rosa et al. [35]. Although HEV3 is considered to be asymptomatic in specific consumers, it has caused a consistent number of infections in which patients declared nausea during the anamnesis step that is usually associated with diarrhea, resulting in its underestimation due to difficultly distinguishing it from other orofecal viral pathogens [11].

Conversely, it may symptomatically infect immunocompromised or immunodeficient patients who require blood transfusions; the consequential risk is also represented by HEV seropositive blood donors who can easily transfer HEV infectious virions, as observed in the Infectious Disease Unit at L’Aquila (Abruzzo region, Italy) by Spada et al. [36]. Finally, it is also mandatory to focus on the cardinal concept that HEV RNA detection does not directly indicate conserved viral vitality or its consequent infectious ability in mammalian hosts. This last point must be considered in order not to spread alarming information [37].

In terms of geography, no evidence of HEV RNA detection was observed in other similar epidemiological studies performed in Europe, such as those of Vercouter et al. [15] in Belgium and Baechlein and Becher [16] among dairy cattle in Germany.

On the other hand, the observed percentage (2.27%) was lower than the 12.30% described by Demirci et al. [14] in Turkey. From a global perspective, high HEV-IgG seroprevalence (about 62.7% of the screened ovine subjects) was observed by El-Daly et al. [38] in Saudi Arabia. They registered the highest prevalence value compared to other animal species (such as cows and goats), providing a scientific epidemiological explanation related to Saudi Arabia’s classification as an endemic HEV geographical area.

These differences and the obtained prevalences are likely associated with differences in the applied farming methods. In further detail, positive ovine raw milk and serum samples, observed by Demirci et al. [14] and El-Daly et al. [38], were correlated with HEV RNA detection in other animal species (bovine, caprine ones, etc.) that are traditionally mixed farmed. Small and traditional rural mixed farms populated with receptive species play a crucial role in HEV RNA persistence and diffusion, as described in the Asiatic and African continents [34,39,40]. Another decisive aspect that can influence HEV RNA detection is the collection moment during lactation [10]. Indeed, it was found that the lactation peak and the viremia phase are responsible for consistent HEV excretions from mammary glands, as comparatively observed in human breast milk [11]. The biochemical explanation for increased host receptibility to HEV virions is related to high estrogen hematic levels, which are associated with a wide synthesis of syndecan receptors expressed by many cytotypes, as explained by Singh et al. [12].

Based on the vital role of the lactation peak in HEV RNA excretion, all animals in the present study were sampled during their peaks (as described in the Section 2).

In Italy, HEV RNA has never been amplified in ovine raw milk before. Furthermore, data on its circulation among the Italian sheep population were collected by Sarchese et al. [17] from fecal and sera specimens at the following percentages: 10.40% and 1.60% (IgG), respectively. In terms of location, the positive milk specimens originated from sheep farmed in the Pescara (H2) and Teramo (H3) provinces were discovered at the following percentages: 3.51% (CI 95%: 0.09–6.93%) and 2.86% (CI 95%: 0.01–5.71%), respectively. The L’Aquila herd (H1) did not harbor HEV RNA in the present study, in contrast to the study by Sarchese et al. [17] on ovine feces in the same province. Furthermore, environmental sharing with wild boars, a viral HEV3 target reservoir [1], represents a scientific demonstration of this pathogen’s viral persistence in a specific geographical area. It is also important to consider that viral fecal elimination covers a wide time range between 2 and 3 weeks from infection. More specifically, the liver and gall bladder (bile excretion) represent long-term viral reservoirs, so intestinal elimination consequently justifies its excretion, as suggested by Ahmed and Nasheri [41].

The pleomorphic environmental behavior of HEV justifies its definition as an emerging and remerging foodborne pathogen [7]. From a food safety perspective, HEV is susceptible to thermal treatments, especially boiling [2,3]; however, the initial viral load plays a defining role in pasteurization efficacy. Indeed, the effects of food technologies (both traditional and innovative) are poorly investigated, and no specific evidence has been produced. The adaptative measures adopted by this viral *noxa* permit its survival and persistence in many food production chains of animal origin, especially in small rural homemade foodstuffs such as fresh liver and meat sausages, salted and seasoned salami, etc. [6]. The different crossing points between domestic and wild receptive animal species, further bolstered by environmental sharing, can be considered crucial aspects, particularly in traditional productive ecological niches. These are widely diffused in many Italian regions, as observed with wild boars [5].

Unpasteurized milk and associated processed products have demonstrated a propensity to harbor HEV RNA, consequentially posing sanitary risks to consumers. Furthermore, the screened ovine populations farmed using the transhumance method showcase traditional animal production methods that characterize Mediterranean Europe, including Italy. The discovered HEV RNA quantities—5/220 or 2.27% (CI 95%: 0.31–4.23%) in tested ovine milk samples, and particularly 3/105 or 2.86% (CI 95%: 0.01–5.71%) in H3 and 2/57 or 3.51% (CI 95%: 0.09–6.93%) in H2—demonstrate HEV RNA circulation in the screened Abruzzo region, as schematically detailed in Table 2 of the Section 3. The harboring of ORF1 and ORF2 highlights and enforces the fundamental role of biomolecular investigation as a surveillance method in these situations of environmental peculiarity. Although the analyzed samples were raw, the virucidal effect of pasteurization still lacks scientific evidence; indeed, its efficacy fundamentally depends on the initial viral loads of milk specimens, as suggested by Huang et al. [34]. New food safety frontiers against viral pathogens derive from innovative technologies such as high-pressure processing and focused ultrasound, which have demonstrated high virucidal effects, particularly on norovirus, as reported by DeWitt et al. [42] and Mustapha et al. [43]. Efficacy against HEV is still lacking; therefore, this gap can be filled through further ad hoc investigations. Although there are still no established legislative limits on HEV quantity (as in the EU Reg. No. 2073/2005), it is highly recommended to strictly apply so-called good hygiene practices, as described in the EU Reg. No. 852/2004. The latter point is necessarily associated with surveillance plans that involve both food operators and public health authorities, focusing on the risk-based assessment approach.

Based on these considerations, further analogous investigations are necessary in order to better clarify the real risk to humans (especially immunocompromised individuals, e.g., transplant recipients, HIV-infected patients, etc.), alongside the impact of traditional and innovative food technologies on HEV virions.

## 5. Conclusions

Three different herds, farmed traditionally following the transhumance method, were bimolecularly screened for HEV RNA. The farming areas included three different national parks (Gran Sasso, Maiella, and Sirente Velino) located in the Abruzzo region (Central Italy). HEV RNA quantities of 10^2^ GE/g were found in positive ovine unpasteurized milk specimens from two different provinces, Pescara (H2) and Teramo (H3), representing potential sources of human infection, especially in immunocompromised patients.

This study confirms the persistence of HEV in the environment, especially in ecosystems shared by multiple species. Traditional, small rural productions also comprise ideal ecosystems for HEV diffusion. This sanitary risk also highlights the importance of producers upholding preventive guidelines in order to reduce and prevent viral circulation in different environments.

Currently, European legislation does not include viral pathogens among the so-called food safety criteria and food hygiene process, as differently described for bacterial *noxae* in the EU Reg. No. 2073/2005. This legal gap shines attention on this significant pathogen (HEV) and others (such as hepatitis A virus, norovirus, etc.), and on the consequential risk to consumers, particularly unpasteurized milk consumers. In addition, the material effects of pasteurization on viral loads (expressed as GE/mL) remain unclear [1]. Based on this legal gap, the precaution concept should be applied, as reported in EU Reg. No. 178/2002, and, as such, boiling procedures or prolonged time/temperature ratios should be considered by food operators.

Following this reasoning, the necessity of improving knowledge on both classical and innovative food technologies emerges. The latter methods (high-pressure processing, focused ultrasound, etc.) have shown consistent applicability in food industries, and their involvement could represent critical control points where food operators can manage different microbiological hazards. Although the present study specifically focused on the Abruzzo region (Central Italy), these methods are also useful for helping local public health authorities understand the epidemiological patterns of particular geographical areas. It is also important that further investigations, involving a wide population, aim to provide data on HEV RNA circulation in Italy.

## Figures and Tables

**Table 1:** Distribution of the ovine herds located in Abruzzo region involved in the present study.

Total Screened Animals	Herds	Abruzzo Province	Screened Animals/Herd
220 Apennine breed sheep	H1	AQ	58
	H2	PE	57
	H3	TE	105

H: herds.

**Table 2:** Obtained results of ORF and GE/mL determinations from ovine raw milk samples.

Positive Animals	Animals and Herds	ORF Detection	GE/mL
5/220 or 2.27%	0/58 or 0.00% H1	-	-
	2/57 or 3.51% H2	ORF-1; ORF-2	10^2^ GE/mL
	3/105 or 2.86% H3	2/3 ORF-2	10^2^ GE/mL
		1/3 ORF-1; ORF-2	10^1^ GE/mL

H: herds; ORF: overlapping open reading frames; GE: genome equivalents.

**Table 3:** Geographical distribution: registered GenBank sequences and herd localizations.

GenBank ID	Herds	HEV Target Genes	Collection Days	Localizations
PQ043352	H2	ORF1	12 February 2024	Vicoli (PE) 42.20 N; 13.54 E
PQ043349		ORF2	17 February 2024	Villa Celiera (PE) 42.22 N; 13.51 E
PQ043353	H3	ORF1	12 February 2024	Valle Castellana (TE) 42.44 N; 13.30 E
PQ043350		ORF2	3 March 2024	Padula (TE) 40.20 N; 15.39 E
PQ043351			8 March 2024	Villa Tofo (TE) 42.39 N; 13.38 E

GenBank ID: identification of registered sequences on GenBank; H: herds.

## Data Availability

The original contributions presented in the study are included in the article, further inquiries can be directed to the corresponding author.
